# Reviews and Book Notices

**Published:** 1880-02

**Authors:** 


### Reviews and Book Notices.

ARTICLE XIV.—CLINICAL MEDICINE; A SYSTEMATIC TREATISE ON THE DIAGNOSIS AND TREATMENT OF DISEASES. Designed for the use of students and practitioners of medicine. By Austin Flint, M.D., Prof. of the Principles and Practice of Medicine, and of Clinical Medicine in the Bellevue Hospital Medical College, etc. Philadelphia: Henry C. Lea. 1879.

This is a new work from one of the best known and most eminent writers on practical medicine in this country. It is a full-sized octavo volume of nearly 800 pages, published in the usual good style of the well-known medical book publishing house of Henry C. Lea. As stated by the author in the preface, the work is devoted almost exclusively to the diagnosis and treatment of diseases, including very little concerning their etiology, special pathology, or morbid anatomy. It seems to have been the purpose of the author to place in the hands of the student and practitioner a work that would aid him in the simple matter of diagnosis and treatment, which he impliedly regards as embracing the special field of clinical medicine. Notwithstanding our high estimate of the author and the value of his many contributions to medical literature, we fail to see the propriety of thus restricting *clinical medicine* to the two topics just named. True, it is the first duty of the practitioner at the bedside of his patient to make a diagnosis, *i. e.*, to determine the name of the disease, its location, extent, and stage of advancement, but does this alone qualify him to prescribe the proper remedies? Is it not just as necessary that he study and see clearly the nature of the morbid action set up and its tendencies; in other words, the special pathology of the case? And how can he apprehend this fully, without reverting to the causes and circumstances under which the disease has been developed. The relations between diagnosis, special pathology, and the indications for treatment, are so intimate that they cannot be separated and maintain a claim to rational practice. The clinical teacher who should occupy the attention of his pupils altogether with the methods of diagnosis and the various remedial agents found valuable in the management of diseases, might make them skillful diagnosticians, but very deficient practitioners. Our author fully acknowledges this, when he says in the preface to the present volume: "The work is intended to accompany, and not, in any measure, to supercede, more comprehensive treatises embracing the morbid anatomy, the causation and the pathology of diseases. It is needless to say that no one who neglects these fields of study can become an intelligent or competent physician; etc." And yet hundreds of students and practitioners will take this new work, restricted as it is to diagnosis and treatment, both as a text-book and a book of reference, and *not* supplement it with more comprehensive works until their modes of thought have become narrowed and partial, and their practice routine and empirical.

We have already too many aids and helps, hand-books and abridgements, summaries and essentials, all tending to encourage superficiality in study, imperfect mental discipline, and still more imperfect professional attainments. We regret that so eminent an author and teacher as Prof. Flint, should have lent any encouragement to this tendency of the times, by giving us a volume in which the important topics of diagnosis and treatment, are so far separated from their intimate and natural relations to pathology and etiology. Having expressed thus freely our disapproval of the general scope of the work so far as it relates to the separation of diagnosis and treatment from the other equally important items of study at the bedside, we turn with pleasure to a brief examination of the contents of the work.

It opens with an introductory chapter of over forty pages containing brief, but excellent instructions concerning the modes of examining patients; the keeping of clinical records and reports; methods of diagnosis and the relations of symptomatology thereto; the objects of treatment both hygienic and medical; the sources of error in therapeutics; and the professional conduct of physicians. Passing this introductory chapter, the author enters directly upon the consideration of the diagnosis and treatment of individual diseases. He follows the general custom of dividing all diseases into two classes, general and local.

He treats of the latter class first, and groups them in accordance with the physiological systems of organs, "namely, the respiratory, circulatory, digestive, urinary, and nervous." A section is devoted to each of these six groups, prefaced by some preliminary observations concerning the means used as aids in observing and properly appreciating the diagnostic symptoms presented at the bedside. These six chapters occupy over six hundred pages, or six-sevenths of the whole volume, leaving only about one hundred pages for the consideration of general diseases. In this latter class, however, the author includes only the idiopathic fevers with diphtheria, erysipelas, gout, rheumatism and epidemic cholera; while chlorosis, pernicious anæmia, Addison's disease, Hodgkin's disease, leucocythemia, general dropsy, pyæmia, septicæmia, scorbutus and scrofula, are classed with the local diseases in the chapter on diseases of the circulatory system. The propriety of such a grouping appears to us very questionable, though of little practical importance. Throughout the volume, in whatever relates to diagnosis and the means and methods to be employed, the author's directions are clear, full and eminently judicious.

We cannot make the same unqualified remark in regard to that part of the work devoted to the treatment of diseases. While the enumeration of remedies is sufficiently full in the great majority of cases, the careful reader, and especially the inexperienced practitioner, anxious for exact knowledge, will notice in reference to many diseases a failure to designate the adjustment of the remedies to the several stages in the progress of diseases under treatment. Take the following as an example, from pages 773–4, in reference to the treatment of acute gout.

"The objects of treatment are to relieve the patient as speedily as possible, and shorten the duration of the attack. Much can be done therapeutically to effect these objects.

"The severe pain should be relieved by some one of the preparations of opium. The promptness of action when administered hypodermically renders the method preferable if, as is usual, the pain be extremely intense.

"If administered by the mouth, the *Dover's* and *Tully's* powders are eligible preparations, in consequence of their diaphoretic effect.

"If the stomach be irritable, laudanum may be given per enema. The doses are to be graduated by the pain, and should be sufficient to afford relief without, of course, incuring any risk of narcosis. A watery extract or infusion of opium may be applied to the effected joint. For shortening the duration of the attack, colchicum is emphatically the remedy. It has no succedaneum in this disease. It should be given as soon as practicable after the paroxysm, that is, at the beginning of the remission or intermission. Half a drachm of the tincture or wine of the colchicum seeds may be given at a dose, and repeated after intervals of three hours, until a moderate cathartic effect is produced. The efficacy of the remedy is not derived from its cathartic effect, but the latter is evidence that it has been given to a sufficient extent."

Here we are told, substantially, to use some one of the preparations of opium to relieve the intense pain of the paroxyism, that we must use it in sufficient doses to accomplish the result, and must be careful not to *narcotize* the patient.

But not one word is said as to what doses of any one of the opiate preparations, an adult patient in the midst of a paroxysm of acute gout, generally requires, or how often it may be safely repeated. And yet that is the very item of information which the student and inexperienced practitioner needs, when he comes to the bedside of his suffering patient. Again, if colchicum is emphatically the remedy for cutting short the attack, why delay its use until the paroxysm subsides? Why not give it in efficient doses conjointly with the opiates; more especially as its efficacy does not depend on its cathartic action?

In our own experience, we have found nothing that would more speedily allay the intense pain of the paroxysm, than an equal mixture of acetated tincture of opium and wine of colchicum seeds, given in doses of from thirty to sixty minims every one or two hours until relief began to be felt, when the interval between the doses is increased.

Again, on page 770, the author writing of the treatment of chronic rheumatism, or the rheumatic diathesis, says:

"While it must be admitted that treatment very often fails, the measures proving in some instances useful, if not successful, are those which have for their object the removal of a diathetic condition. Various medicinal remedies are serviceable, each in a certain number of cases, and, and with proper caution, they should be severally tried before coming to the conclusion that nothing is to be expected from drugs. The following are the more important of these remedies: guaiacum, mercury in the form of bichloride or the iodide, the iodide of potassium or ammonium, colchicum in small doses, the hydrochlorate of ammonia, arsenic and sulphur."

Here are eight or nine efficient drugs named, differing widely from each other in composition, and mode of action upon the human system, and it is intimated that each one of them is capable of benefitting "a certain number of cases" of chronic rheumatism; but no attempt is made to point out the particular kind of cases that each remedy is most likely to benefit. On the contrary, the student is rather plainly encouraged to begin the list with each unfortunate patient, and proceed blindly from drug to drug until the desired relief is obtained or the list has been completed. It is true, the author says this should be done "with proper caution."

But what constitutes proper caution in the use of these remedies in the treatment of chronic rheumatism? Does it consist in stopping when the *gums are touched* by the mercurials; the bowels purged by the colchicum; or the appearance of initial symptoms of general dropsy from the arsenic? These are certainly questions of interest to the practitioner, and such as a full treatise devoted exclusively to diagnosis and treatment should answer. We have quoted the above simply as specimens of what may be found in almost all parts of the volume before us. They certainly very much lessen the value of the work, especially as a text-book for students. The same defects are found more or less, however, in most of the more recent works on practical medicine.

It is not uncommon to find forty or fifty pages devoted to a consideration of the symptoms, causes, pathology, morbid anatomy and diagnosis of a disease, while the treatment is summarily disposed in one or two pages.

This is not as it should be. The real end of all our study is to learn the art of prevention and cure of diseases.

And our success will always depend fully as much upon the accuracy with which we adjust our remedies to the stage of progress, and the special character of the morbid action in each case, as upon the kind of remedies we choose. N. S. D.

ARTICLE XIV.—THE PATHOLOGY AND TREATMENT OF VENEREAL DISEASES. By Freeman J. Bumstead, M.D., LL.D., late Professor of Venereal Diseases at the College of Physicians and Surgeons, New York, etc., etc. Fourth edition, revised, enlarged and in great part rewritten by the author and by Robert W. Taylor, A.M., M.D., Professor of Skin Diseases in the University of Vermont, Attending Surgeon to Charity Hospital, etc., etc. With one hundred and thirty-eight woodcuts. 8vo. pp. 835. Philadelphia: Henry C. Lea. 1879.

The first edition of this familiar work, issued in 1861, commanded a success which was the fruit not only of its intrinsic excellence, but of its timeliness. It was presented to the profession of this country before the time of that remarkable awakening of interest in venereal studies, to which the book itself in large part contributed. At a date when the subject had been largely relegated to the great teachers of surgery in America, who for the most part, it may be said, viewed it from the standpoint of so-called unicism, this treatise, written in a remarkably lucid and pleasant style, attracted universal attention to the doctrines of the two men, who were then the rising and setting suns of French syphilography. The book thus attained a popularity among medical men, the like of which can scarcely be realized a second time, either by itself or its competitors, from the lack of the peculiar timeliness which has been already mentioned. In brief, the work became the oracle, in its special field, for many who read its pages with an interest which was only equalled by their delight. Nor was this true only of professional gentlemen.

Venereal patients in the *clientèle* of the general practitioner, purchased and perused it, with an eagerness born of their necessities, and even quoted its precepts to those in whose charge they had sought relief. Beyond the borders of the United States, such was its popularity, that before the issue of a second edition it received the honor of Italian translation and publication.

The editions which followed, were revised and improved to a certain extent, sufficient at least to sustain the high reputation of the treatise; but the later, it must be confessed, were threatened with eclipse by the appearance of a new and excellent work, which was also from the pen of American authors of experience and well-known ability, and which rapidly commanded the position to which it was entitled. Certainly it was high time for a more thorough and complete revision of this volume, which, more fully than any other, has become identified, on this continent at least, with the literature of venereal diseases. The prestige of a large and deserved success could not much longer withstand the eager competition of a host of American authors and observers of unquestionable merit.

The task has been accomplished, and that right worthily. We find that we have here practically a new book, and that the statement of the title-page as to the fact that it has been largely rewritten, is a sufficiently modest announcement of the important changes in the text. Dr. Taylor, who was selected to aid in the work of revision, is already favorably known to the profession at large as an author of numerous monographs on dermatological and venereal subjects, not the least of which is his valuable essay on "Syphilitic Lesions of the Osseous System in Infants and Young Children," New York, 1875; a work which has gained for the writer an international reputation in this special department of a special branch of medicine. It is not difficult to detect his careful handiwork in the transformation of the treatise.

How great this task has been, will best be determined by him who has made the attempt to examine, even in a cursory fashion, the literature of any other medical subject for a single year. From January to November, 1879, for example, no fewer than 394 papers, pamphlets, books, articles in medical periodicals, monographs, etc., were printed under the designation of syphilis and gonorrhœa only, and to this large number should be added, for the current year, not only those to appear during the last two months, but an equally large, if not still greater number of titles in the department of skin diseases, general pathology, general surgery, and genito-urinary disorders. All these, appearing in the English, French, German, Italian, Spanish and Russian languages, furnish a truly formidable mass of material, which accumulates between the dates of the several editions of any given work, and much of which should pass under the eye of the writer of a systematic treatise on venereal diseases. For the day has passed, rarely to return, when originality of experience or expression can take the place of this laborious compilation, digestion and condensation of the labor of the best men. It has nearly come to this, that in the day which we see and in the departments of science we cultivate, the less original and the more catholic a book, the greater its chances of success and survival. To return to the volume before us, it is not too much to say that a still larger treatise could have been filled with what is to-day known about either nervous, hereditary or osseous syphilis, stricture, the diseases of the prostate gland due to gonorrhœa, or a dozen other topics which might be named.

We have made this explanation, chiefly because, after a thorough examination of the present edition, we can assert confidently that the enormous labor we have described, has been here most faithfully and conscientiously performed. They who know how to appreciate the difficulty of such a task, will be surprised, when they come to consult these pages for the literature of any important subject, to discover how far an abstract of the same has been incorporated with the text.

A few illustrative examples will suffice to direct attention to this valuable quality of the work accomplished. Hydrocele, for instance, is not a subject which has a direct connection with venereal disorders, and on turning to the latest edition of Mr. Curling's voluminous treatise on the testis,* we find 132 pages devoted to the thirteen varieties and complications of hydrocele there tabulated. But our authors have succeeded admirably in conveying

* London, 1878.

all essential information on this topic, in the closely-printed six pages which are by them allotted to the subject.

A comparison of the book before us with one of the latest published under a similar title in France, will serve to establish the fact of the relative excellence of the American treatise. The comparison is the more justifiable, as our American authors have not hesitated to make frequent references to the labors of M. Louis Jullien,* and even to borrow some of his plates.

On the question of the etiology of hereditary syphilis, which has of late elicited a great deal of discussion, and which has attracted the attention of eminent men, who have espoused every succeeding solution to the problems presented, the differences between the writers in comparison become conspicuous. Jullien, resting implicit faith upon the hitherto impregnable law, formulated by Colles in the year 1844, that a child affected with hereditary syphilis will not infect the breast of the mother who suckles it, hastens to set forth the following propositions as fully established: 1. Without infection of the mother, there can be no hereditary syphilis. 2. The father cannot transmit syphilis to his child without contamination of the mother.

But these propositions have been refuted, time and again, by clinical observations of the most scrupulous exactness, extending, as is requisite, over long periods of time, and conducted under the serveillance of scientific men of established skill and probity. The results are fixed facts, which are not to be either neglected or explained away. But there is a peculiarity in the mental constitution of French medical authors, which is an inextinguishable reverence for Gallican ideas and Gallican heroes. The doctrines which Jullien sustains are the untenable theories which Cullerier first asserted, which have since borne his great name, and which were shown to be false and dangerous even before the publication of the masterly monograph by Kassowitz, which, if it proved nothing else, showed that in the paths of brilliant and original investigation, the sceptre was departing from the French nation.

Upon the points in question, Bumstead and Taylor express opinions which are in accord with the views of the most judicious authors in this department. Syphilis, say they, is transmissible

* Traité Pratique des Maladies Vénériennes, Paris, 1879.

from the father to his offspring. Viewing the law of Colles in the only light which is warranted in the present state of our knowledge, they declare that the immunity of the mother of the infant who has inherited syphilis, is due to some occult and undiscernible change in her system. They refuse to declare that such immunity is equivalent to maternal syphilis, until that syphilis is pronounced and demonstrable. This is really not merely a curious problem for the theories of doctrinaires. It is a practical and every-day question of pressing importance. Every syphilitic patient has the right to know himself or herself to be such, and it is for the interest of each as well as for the interest of society at large that such knowledge should be had. What refinement of cruelty is greater than that which suspends a sword worse than that of Damocles, for the space of years (and there are cases where this period has extended to a quarter of a century), over the head of one who should enjoy the fullest assurance of safety!

Again, with regard to the *choc en retour* of Ricord, and the fanciful "syphilis by conception," more recently advocated by Diday, of Lyons, it seems well-nigh impossible for Jullien to divest his mind of an extraordinary sentiment of reverence for him whom the French are fond of calling the "Lyonnaise master." Jullien declares that Diday's method (of regarding the menstrual derangements of the wife of a syphilitic husband as so many abortions with syphilitic ova) is exceedingly ingenious, and one which supplies the key to a great many problems. He proceeds to express his admiration for the way in which Diday has expounded his theory. At the same time, the opinion is advanced that the theories in question are not such as to command absolute conviction; and a mild request for further proof is made, accompanied by an apology for taking such a liberty.

Now the fact is, that Diday's absurd paper on this subject has already been shown to contain errors and damaging omissions. It has not, so far as we are aware, received the indorsement of a single syphilographer of repute; and its quasi support by Jullien is, to us, an additional evidence of the prevailing French method of writing books in which everything that emanates from the pen of a French writer is regarded with at least respectful consideration, while the views of those who oppose the latter, provided the authors are not of French nationality, are esteemed as those of "barbarians," in the Greek acceptation of that word.

Bumstead and Taylor have the courage to pronounce Diday's recent article as "valueless and defective," and formulate the conclusion to which they have arrived as follows. It is in accord with what seems to us the most judicious interpretation of established facts: "We conclude that, in hereditary syphilis, the disease is conveyed either by the sperm-cells or by the ovule, diseased at the time of conception, and that infection of the mother or of the child cannot take place through the utero-placental circulation."

Lastly, a further comparison of the two books serves to exhibit the care which has been bestowed upon typographical appearance by the Americans. They furnish a list of twelve errata, printed on the page which faces the opening sentence of the introduction; and the character of these is such as to require, in the main, a change of but one letter or the addition of an *umlaut*. The errors belong, in short, to that list which should be recognized as the "inevitable" in the prosecution of so great a task as the publication of a treatise of this size and scope. The proof-reading has evidently been of the most conscientious character, and has thus contributed in no small degree to the pleasure which the perusal of the work affords.

In Jullien's work, however, can be recognized that sublime indifference to the orthography of all languages other than the French, which observant men have come to regard as a distinctive feature of their scientific publications. We have enumerated no less than thirteen typographical errors in two successive pages of bibliography given in the "Maladies Vénériennes," and it is fair to infer from this that are more than one thousand in the entire 1120 pages. It should be added that these errors are not only those which occur in the rendition of foreign names; we note these also in the spelling of French words, and even in the titles of pages.

We have endeavored thus briefly to suggest some comparison between these two works, lately issued on the same subject, that the results might indicate more clearly than could the mere dictum of a reviewer what reason we have to congratulate our countrymen upon the truly valuable addition which they have made to American literature. The careful estimate of the value of the volume, which we have made, justifies us in declaring that this is the best treatise on venereal diseases in the English language, and, we might add, if there is a better in any other tongue, we cannot name it; for, while it may be admitted that there are to be found elsewhere, on certain special subjects, fuller discussions of every phase of controverted themes, there are certainly no books in which the student or the general practitioner can find such an excellent résumé of the literature of any topic, and such practical suggestions regarding the treatment of the various complications of every venereal disease.

We note that the metric system has been so far adopted by our authors that in all printed prescriptions the dose is named both in terms of the old and new nomeclature, an arrangement which has been adopted by several German authors since the use of the metric system was in their country made imperative by law. The method of our authors is one which should satisfy the friends and enemies alike of the recommendations of the American Medical Association in the same connection. The prescriptions of our authors are, as a rule, too, metrically accurate, and not mere transpositions of terms. A few errors, in the accomplishment of this task, were only to be expected; and we note one on the 573d page, where a formula is given for the treatment of phtheiriasis pubis. The proportions are accurately given in terms of the metric system, but, by the old reading, the solution of the bichloride of mercury ordered will contain sixteen grains to the fluid ounce. This solution would, without any question, put an end to the lice, but we may be permitted to express the hope that the tyro who first consults the page for the purpose of being instructed as to what he shall employ, will prefer to be guided in his selection by the proportion given in the metric formula.

In the matter of mensuration of urethral instruments, our authors have also, in the present edition, given a deserved preference to the French scale; figured the Handerson gauge, which we esteem far more useful than any others sold (even to the exclusion of the lately-devised "adaptable metric gauge" of Dr. C. H. Thomas, of Philadelphia); and have, in our judgement, done well in inclosing within quotation marks the name of the so-called "American scale," proposed by Drs. Van Buren and Keyes, in their otherwise admirable treatise. If these last-named gentlemen had been content with baptizing their scale with their own names, an immense deal of confusion would have been avoided. By attaching a national name to their scale of instruments they accomplished a result which we can well believe they did not anticipate. They might have been speedily forgiven for the boldness of their action in this matter, if it had resulted in giving us a truly American uniformity. As it is, we are still compelled to follow our authors in the adoption of that which "is known and used as a standard by so many surgeons in every civilized country."

In the matter, too, of the use of instruments for mensuration of the urethral chink, when it is distended, and for incision of strictures of large calibre, we congratulate our authors upon the judicious fairness with which they have handled the subject. They have not failed to give honor where honor was due, and, refusing, practically, to embody a section of a controversy in their pages, which would only serve to disfigure them, they have earned for themselves a merited commendation.

The style of the book, as re-written, is fully equal to that of the preceding editions, which was in parts a model of elegant English. It must be admitted, however, that a species of incongruity can be detected, in the indiscriminate use of the personal pronoun, which leaves the reader occasionally in doubt as to whether the senior or the junior author is responsible for the text, or whether they have both contributed to its lines. It would surely have been an improvement if the editorial "we" had been adopted throughout, as thus the unity of the book would have been more completely sustained.

It is sufficient to ask of our authors in medicine that they embody in their work, the fruit of the researches of the most trusted of their contemporaries and predecessors. It is too much to ask of them that they also point out to us the lines of probable advance in the wilderness of the knowable and yet unknown or but imperfectly known. We can, therefore, well afford to excuse the writers of the treatise before us, for their halt in the face of a proposition to which it seems to us the scientific world must speedily give assent. It is the proposition which asserts the contagiousness, in different degrees, of all pus. Or, to place the doctrine in a clearer light, it is a proposition which asserts that the living product of the process of inflammation is possessed of an energy which is capable of originating a like process in all living matter, this capability being proportioned to the activity of the process by which it was itself engendered.

First announced by Von Roosbroeck, we believe, and substantiated by experiments by Pick, of Prague, in so far as it relates to the matter of the acne pustule, it is an explanation of much that is either doubtful or curious in pathology. It may be found to throw some light on the "contagious impetigo," of the late lamented Tilbury Fox, and, if finally well established, will splendidly illuminate many obscure points in the study of venereal diseases. If all pus is more or less contagious, we may be sure that the pus produced by the inflammatory process in venereal disorders, will enjoy its full share of such a power. We shall not then find it to be absolutely true, as stated by our authors (p. 398), that "pus of a sinple inflammatory bubo is like that of any common abscess, destitute of contagious properties, and therefore not inoculable." For it is precisely the pus of a common abscess which we shall one day find to possess the exact qualities which are here denied to it, unless all the sign-posts, which are now plainly pointing in that direction, be speedily removed.

But as we have said, it is altogether too much to require of our standard text-books that they be not only encyclopædias of wisdom, but oracles of prophecy as well.

The several plates in the work are all finely finished, and the reader will be pleased to find here many that are not only entirely new, but of great service in the explanation of the text. The publisher has not failed to execute his part of the labor of introducing this practically new work, in an exceedingly handsome dress. The volume is larger than its predecessors by 131 pages, but from a reduction in the size of the type, it is estimated that it contains about twice as much matter. The index to this edition, we find full and accurate. In the matter of the abbreviations of the titles of periodicals, the plan has been here followed, which was first employed by Dr. Billings, of the library of the Surgeon-General's office.

We take pleasure in repeating that we believe this to be the best treatise on venereal diseases in the English language, and we congratulate the authors upon their brilliant addition to American medical literature.

The news of the death of Dr. Bumstead, which occurred on the 28th of November, 1879, and which attaches a melancholy interest to this latest work of his pen, reached us only after the completion of the preceding paragraphs. Having written in an effort to impartially consider the fruit of his labor, we have preferred to make no change in any sentence. Needless to say, however, that if we could have known the learned author was to survive for but so brief a time the exhausting labor which the preparation of his book enforced, we should have approached the task of reviewing it with greater diffidence and turned its pages with a gentler hand.

Others, without doubt, will pay the timely and tender tribute to that intellectual manhood, which is hereafter to adorn the roll of the honored dead of the profession of our country, and to those genial qualities of heart which have endeared their owner to his many personal friends. We can not but be grateful that his life was spared to add the finishing touches to this great work, in which all his largest activities were interested, and which will survive as a fitting monument to the brilliant success of his professional career. We would lay this as a flower on the tomb of the dead master, that in a field which others might esteem repulsive, he wrought that which has the fairness and purity of the spirit by which he was moved.

J. N. H., *in the American Journal of the Medical Sciences.*

